# Mitoferrin-1 Promotes Proliferation and Abrogates Protein Oxidation via the Glutathione Pathway in Glioblastoma

**DOI:** 10.3390/antiox12020349

**Published:** 2023-02-01

**Authors:** Md Yousuf Ali, Corinne E. Griguer, Susanne Flor, Claudia R. Oliva

**Affiliations:** 1 Interdisciplinary Graduate Program in Human Toxicology, The University of Iowa, Iowa City, IA 52242, USA; 2 Free Radical & Radiation Biology Program, Department of Radiation Oncology, The University of Iowa, Iowa City, IA 52242, USA

**Keywords:** glioblastoma, 4-HNE, iron, mitoferrin, Michael adducts, glutathione

## Abstract

Median overall survival is very low in patients with glioblastoma (GBM), largely because these tumors become resistant to therapy. Recently, we found that a decrease in the cytosolic labile iron pool underlies the acquisition of radioresistance. Both cytosolic and mitochondrial iron are important for regulating ROS production, which largely facilitates tumor progression and response to therapy. Here, we investigated the role of the mitochondrial iron transporters mitoferrin-1 (MFRN1) and mitoferrin-2 (MFRN2) in GBM progression. Analysis of The Cancer Genome Atlas database revealed upregulation of MFRN1 mRNA and downregulation of MFRN2 mRNA in GBM tumor tissue compared with non-GBM tissue, yet only the tumor expression level of MFRN1 mRNA negatively correlated with overall survival in patients. Overexpression of MFRN1 in glioma cells significantly increased the level of mitochondrial iron, enhanced the proliferation rate and anchorage-independent growth of these cells, and significantly decreased mouse survival in an orthotopic model of glioma. Finally, MFRN1 overexpression stimulated the upregulation of glutathione, which protected glioma cells from 4-hydroxynonenal-induced protein damage. Overall, these results demonstrate a mechanistic link between MFRN1-mediated mitochondrial iron metabolism and GBM progression. Manipulation of MFRN1 may provide a new therapeutic strategy for improving clinical outcomes in patients with GBM.

## 1. Introduction

Glioblastoma (GBM) is the most common and most aggressive brain cancer, with a median survival of 14–16 months [1]. The current standard-of-care therapy for GBM includes surgical removal of the tumor, followed by chemotherapy with temozolomide and radiotherapy [2]. Although patients with GBM receive an initial benefit from this treatment, the development of tumor cell resistance to chemotherapy and radiation is one of the major challenges to achieving a sustainable survival benefit in GBM [3]. Better characterization of the molecular mechanisms within glioma cells that promote tumor progression and treatment resistance is necessary to inform the design of more effective primary or adjuvant therapies for patients with GBM.

Extensive research has shown that dysregulation of cellular iron levels and metabolism promotes tumor initiation and growth and determines the response of mammalian cells to radiotherapy, largely by regulating the abundance of reactive oxygen species (ROS) [4,5,6,7]. These ROS are crucial for producing the molecular mediators of cell damage and death, including lipid peroxidation-derived reactive aldehydes, such as 4-hydroxynonenal (4-HNE) [8,9,10,11]. We previously showed that the cytosolic labile iron pool—the portion of iron that is not transferrin bound and thus remains redox active—has a significant role in determining the response of GBM cells to radiation, with a decrease in this pool contributing to radioresistance [12]. This decrease in the labile iron pool is associated with increased activity of cytochrome c oxidase (CcO), a crucial component of the mitochondrial electron transport chain (ETC), and a decrease in ROS production. Considering that mitochondrial iron homeostasis is particularly crucial for the synthesis of the iron-sulfur (Fe-S) clusters and heme necessary for the assembly, stability and function of the ETC complexes [13,14,15], we hypothesized that the mitochondrial import of labile iron is necessary for such effects.

Two mitoferrin proteins, mitoferrin-1 (MFRN1; encoded by the solute carrier family 25-member 37 gene [SLC25A37]) and mitoferrin-2 (MFRN2; encoded by SLC25A28), are the most significant mitochondrial iron importers [16,17] and are critical regulators of cytosol-mitochondria iron homeostasis in human cells [18,19,20,21]. Mitoferrin dysregulation has been implicated in a variety of disorders, including the progression and response to therapy of some tumors [18,19,21,22,23,24,25]. However, the effects of MFRN1 and MFRN2 dysregulation appear to vary by cell type and pathologic condition. In this study, we assessed the potential role of MFRN1 and MFRN2 and mitochondrial iron homeostasis in the progression of GBM.

## 2. Materials and Methods

### 2.1. Gene Expression Analyses

For comparisons between glioma tissue and normal brain samples, MFRN mRNA expression from non-tumor (28 samples) and GBM (219 samples) was obtained from the TCGA Rembrandt dataset. Expression of MFRN mRNA stratified by tumor grade in glioma samples (Oligodendroglioma, 191 samples; Oligoastrocytoma, 130 samples; Astrocytoma, 194 samples; and GBM, 194 samples) was obtained from the TCGA GBMLGG dataset. Kaplan–Meier survival analysis was performed with the TCGA_GBM dataset using median gene expression to split high and low MFRN expression populations. The TCGA database can be downloaded from the GlioVis data portal (http://gliovis.bioinfo.cnio.es/, date accessed 21 September 2022).

### 2.2. Quantitative Real-Time PCR (qRT-PCR)

Total RNA was extracted using an RNeasy Plus RNA extraction kit (Qiagen, Germantown, MD, USA) according to the manufacturer’s instructions, and 2 µg of total RNA was added in a reverse-transcription reaction to generate the first strand of cDNA using a High-capacity cDNA Reverse Transcription Kit (Applied Biosystem, Waltham, MA, USA). The synthesized cDNA was then subjected to qRT-PCR with the following primers (MFRN1: Forward 5′- TAG CCA ACG GGA TAG CTG G -3′; Reverse 5′- GTG GTG TAG CTC CGG TAG AAG -3′; 18S: Forward 5′- ACC CGT TGA ACC CCA TTC GTG A -3′; Reverse 5′- GCC TCA CTA AAC CAT CCA ATC GG -3′). The mRNA expression level of 18S rRNA served as an internal standard control.

### 2.3. MFRN1 Protein Expression Analysis

MFRN1 protein expression levels were determined in mitochondrial extracts by Western blot analysis, as we previously described [26,27,28]; 10 µg of each extract was loaded and electrophoresed in 4–20% TGX mini precast polyacrylamide gels. Proteins were then transferred to polyvinyl fluoride membranes, and the membranes were incubated with anti-MFRN1 (1:1000, #26469-1-AP, Proteintech, Rosemont, IL, USA) and anti-citrate synthase (1:1000, #16131-1-AP, Proteintech) (loading control) primary antibodies overnight at 4 °C. Membranes were incubated with anti-mouse IgG kappa binding protein (1:2000, sc-516102, Santa Cruz Biotechnology, Dallas, TX, USA) or anti-rabbit IgG (1:2000, sc-2357, Santa Cruz Biotechnology) secondary antibody conjugated with HRP for 1 h. Membranes were developed with ECL HRP substrates and exposed to Autoradiography Classical X-ray Film.

### 2.4. Cell Lines and Maintenance of Cell Culture

The U251 human glioma cell line was originally obtained from Dr. G. Yancey Gillespie (University of Alabama at Birmingham, Birmingham, AL, USA) and was authenticated using a short tandem repeat (ATCC, STR service, Manassas, VA, USA). U251 glioma cells were cultured and maintained, as previously described [28,29,30]. The cells were maintained in DMEM F12 medium (Corning, Manassas, VA, USA) supplemented with L-glutamine (MP Biomedicals, Solon, OH, USA) and 7% heat-inactivated fetal bovine serum at 37 °C and 5% CO_2_ in a humidified incubator (Thermo Fisher Scientific, Waltham, MA, USA).

### 2.5. Generation of MFRN1-Overexpressing Cells

U251 cells were electroporated with CMV6 plasmids containing Myc-DDK-epitope-tagged human MFRN1 or the pCMV6-Entry mammalian vector with a C-terminal Myc-DDK tag as the control (NM_016612.4, catalog # RC218413 and PS100001, respectively; OriGene Technologies, Rockville, MD, USA). Cells were electroporated using a Gene Pulser Xcell Electroporation System (BioRad, Hercules, CA, USA) and selected with neomycin (800 µg/mL) as we previously described [29,31]. MFRN1 expression was analyzed in isolated clones by qRT-PCR and Western blot analysis. Two clones with stable overexpression of MFRN1, 9 and 10, were identified from the screening.

### 2.6. Cell Proliferation and Anchorage-Independent Clonogenic Assays

For cell proliferation assays, cells were seeded into 6-well plates (2 × 10^4^ cells/well). The cell number was determined using a TC20 automated cell counter (BioRad). Anchorage-independent clonogenic assays were performed, as we previously described [31]. For these assays, two layers of agarose were prepared in a 6-well cell culture dish. To produce the bottom layer, 2 mL of melted SeaPlaque agarose (0.9% final concentration) and medium mixture (1:2 agarose to medium ratio) were added per well. The mixture was allowed to solidify at room temperature and then transferred to a refrigerator for overnight incubation. To produce the top layer, 2 mL of a mixture of the agarose and culture medium (1:3 agarose to medium ratio) was added on top of the bottom layer of solidified agarose in each well, and 30 µL of freshly trypsinized and resuspended cells (3000 cells) was immediately added. This layer was allowed to solidify for 30 min at room temperature and then incubated at 37 °C for 14–21 days. The colonies were then stained with p-iodonitrotetrazolium chloride (Sigma, Saint Louis, MO, USA). Images of the stained colonies were obtained from individual wells, counted and analyzed using a GelCount imager and software (Oxford Optronix Ltd., Abingdon, OX, UK). 

### 2.7. Xenograft GBM Tumors

The establishment of intracranial tumors was performed as we previously described [29,31,32]. All surgical and experimental procedures and animal care practices were performed in compliance with the policies approved by the Institutional Animal Care and Use Committee of the University of Iowa. Mice exhibiting signs of neurological deterioration were sacrificed, and the brains were removed for analysis. Sections of paraffin-embedded tumor tissues were stained with hematoxylin and eosin (H&E; #ab245880, Abcam, Waltham, MA, USA, Cat.) and Ki67 as a proliferation marker (# ab16667, Abcam), as we previously described [29,31].

### 2.8. Measurement of Mitochondrial Iron, Labile Iron Pool and Lipid Peroxidation

A total of 2 × 10^5^ cells were plated in 6-well plates and cultured for 24 h. Cells were then labeled with 7.5 μM Mito-FerroGreen [33] (Dojindo Molecular Technologies, Kumamoto, Japan) in Hank’s Balanced Salt Solution (HBSS, Thermo Fisher Scientific) for 25 min at 37 °C. Samples were analyzed on a Becton Dickinson LSR II flow cytometer (BD Biosciences, Franklin Lakes, NJ, USA) at 495 nm (excitation) and 515 nm (emission). The obtained data were analyzed using FlowJo v10 software.

The labile iron pool was determined using the fluorescent dye calcein-AM (Thermo Fisher Scientific), as previously described [12].

Lipid peroxidation was measured with BODIPY C11 staining (Invitrogen, Waltham, MA, USA), as we previously described [12].

### 2.9. Measurement of 4-HNE Protein Adducts

The formation of 4-HNE protein adducts was determined by Western blot analysis. Untreated cells and cells treated with 4-HNE (Cayman Chemicals, Ann Arbor, MI, USA; 25, 50 or 100 µM for 1 h), buthionine sulfoximine (BSO; Sigma; 5 mM for 24 h), or erastin (Selleck Chemicals, Houston, TX, USA; 20 µM for 24 h) were scraped and total lysates were prepared using CelLytic M (Sigma). Total lysates (30 µg) were loaded, electrophoresed and then transferred to PVDF membranes. Membranes were incubated in 100 mM MOPS (Sigma; pH 8.0) with 250 mM sodium borohydride (Sigma) for 15 min, washed and blocked, and then incubated overnight with anti-4-HNE Michael adducts antibody (1:2000; #ABN249, Sigma). After rinsing and blocking, the membranes were incubated with anti-rabbit IgG secondary antibodies conjugated with horseradish peroxidase (HRP) (1:2000, sc-2357, Santa Cruz Biotechnology, Dallas, TX, USA) for 1 h.

### 2.10. ETC Complexes and Citrate Synthase Activity Assay

Mitochondria fractions were prepared as previously described [28,34,35,36]. The activity of ETC complexes and citrate synthase was measured in mitochondrial extracts, as previously described [37].

### 2.11. Statistics

All data were evaluated using GraphPad Prism (GraphPad Software, San Diego, CA, USA). Results are expressed as the mean ± SD, and *p* < 0.05 was considered significant. Statistical analyses were performed using one-way analysis of variance (ANOVA), followed by Tukey’s multiple comparison test or an (un)paired Student t-test. Statistical significance was indicated with asterisks: * *p* < 0.05, ** *p* < 0.01, *** *p* < 0.001 and **** *p* < 0.0001.

## 3. Results

### 3.1. MFRN1 Expression Correlates with Poor Patient Survival in GBM

**Figure 1:**
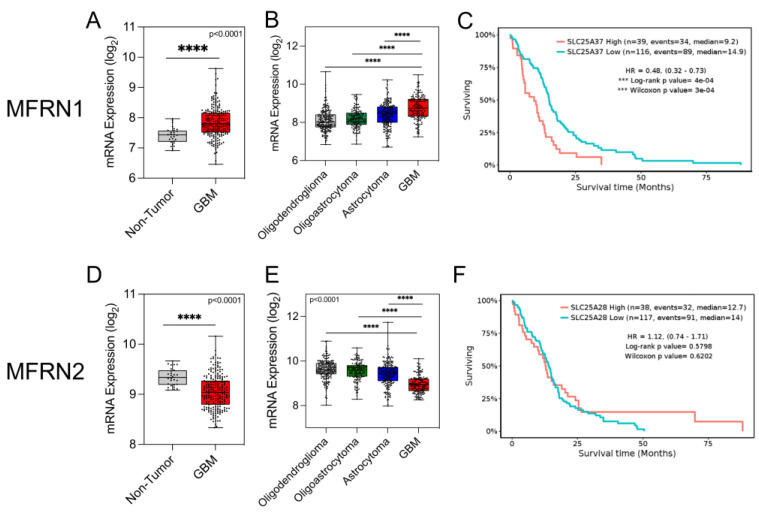
Expression of MFRN1 and MFRN2 mRNA in human GBM. (**A**), Comparative analysis of MFRN1 mRNA expression in glioma tissue and non-tumor brain tissue (Rembrandt dataset). Lines in the scatter plot represent median values. **** *p* <  0.0001 by ANOVA. (**B**), Expression of MFRN1 mRNA stratified by tumor grade in glioma samples from the GBMLGG dataset. Median values are represented as lines in the scatter plot. **** *p* <  0.0001 by ANOVA. (**C**), Kaplan–Meier curves analysis of OS in glioma patients stratified by MFRN1 mRNA expression levels in tumors (low MFRN1, n = 116; high MFRN1, n = 39). *** *p* < 0.001. (**D**), Expression of MFRN2 mRNA in glioma tissue and non-tumor brain tissue samples from TCGA dataset. Median values are represented as lines in the scatter plot. **** *p* <  0.0001 by ANOVA. (**E**), Expression of MFRN2 mRNA stratified by tumor grade from the GBMLGG dataset. Median values are represented as lines in the scatter plot. **** *p* <  0.0001 by ANOVA. (**F**), Kaplan–Meier curves of OS in patients with glioma stratified by mRNA expression of MFRN2 in tumors (low MFRN2, n = 117; high MFRN2, n = 38). HR, hazard ratio.

To investigate the clinical relevance of MFRN1 and MFRN2 expression in gliomas, we interrogated data from The Cancer Genome Atlas (TCGA) accessed via GlioVIS. Compared with the expression levels in non-tumor tissue and in low-grade glioma samples, the expression of MFRN1 mRNA was significantly upregulated in GBM samples (Figure 1A,B). Furthermore, MFRN1 mRNA expression in tumors correlated inversely with overall survival (OS) in the patients (high MFRN1 expression: OS = 9.2 months; low MFRN1 expression: OS = 14.9 months; *p* < 0.0001 by log-rank test) (Figure 1C). In contrast, the expression of MFRN2 mRNA was downregulated in the GBM samples (Figure 1D,E) and did not correlate with a difference in OS (high median MFRN2 expression: OS = 12.7 months; low median MFRN2 expression: OS = 14.0 months; *p* = 0.58 by log-rank test) (Figure 1F). These data suggest that the higher tumor expression of MFRN1 in GBM is associated with a poor prognosis.

### 3.2. MFRN1 Overexpression Promotes Glioma Cell Proliferation

**Figure 2:**
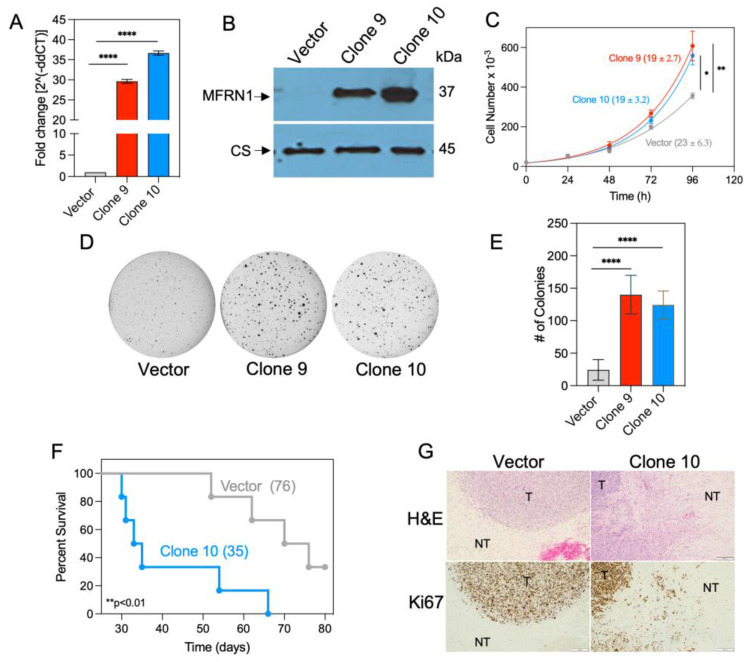
MFRN1 expression in stably transfected glioma cells. U251 cells were transfected with a vector expressing MFRN1 (pCMV6−CAT−-Myc−DDK) or the vector only (pCMV6−Myc−DDK) as a control and treated with G418 to produce stably transfected clones. (**A**), Quantitative analysis of MFRN1 mRNA by qPCR. Graphs represent the mean ± SEM of duplicate determinations from three independent experiments. **** *p* < 0.0001 calculated by Student *t*-test. (**B**), Representative Western blot depicting MFRN1 protein expression in mitochondrial extracts of select clones (9 and 10). Citrate synthase (CS) was used as a loading control. (**C**), Cell proliferation analysis in cell cultures of control and MFRN1-overexpressing cells. (**D**,**E**), Representative images (**D**) and quantitative analysis (**E**) of anchorage-independent colony formation in control and MFRN1-overexpressing cells. (**F**) OS in nude mice harboring orthotopic brain tumors generated by inoculation with vector control-transfected or MFRN1-overexpressing (clone 10) U251 cells (n = 6 per group). (**G**), Representative images of control and MFRN1 tumors stained for hematoxylin and eosin (H&E) and Ki-67. T, tumor; NT, Normal tissue. * *p* < 0.05, ** *p* < 0.01, and **** *p* < 0.0001 calculated using one-way ANOVA followed by Tukey’s multiple comparison test or unpaired, two-tailed Student *t*-test.

To evaluate the effect of MFRN1 overexpression on the glioma phenotype, we stably transfected U251 glioma cells with a vector encoding MFRN1 cDNA (clones 9 and 10) or an empty vector as the control. The expression of MFRN1 was determined by qRT-PCR and Western blot analyses (Figure 2A,B). 

To determine whether MFRN1 overexpression can affect glioma cell growth, we examined U251 cell proliferation. In adherent cell cultures, overexpression of MFRN1 significantly decreased the doubling time (19.7 h and 19.2 h for clone 9 and 10 cells, respectively, versus 24.5 h for vector control U251 cells; *p* < 0.001) (Figure 2C). MFRN1 overexpression also markedly promoted colony formation in anchorage-independent (soft agar) cultures (colony number: 140 ± 8.6 and 124 ± 6.1 for clone 9 and 10 cells, respectively, versus 24 ± 3.6 for vector control U251 cells, *p* < 0.0001) (Figure 2D,E).

To determine whether overexpression of MFRN1 in GBM affects host survival, MFRN1-overexpressing (clone 10) or vector control U251 cells were implanted orthotopically into the brains of nude mice. Median survival was significantly shorter in mice bearing MFRN1-overexpressing tumor cells (35 days versus 76 days in mice bearing vector control tumor cells) (Figure 2F). Notably, extensive tumor infiltration into normal brain tissue was observed in brains from mice bearing MFRN1-overexpressing cells. Additionally, Ki67 immunohistochemistry was performed to examine Ki67 levels, a marker strongly associated with tumor cell proliferation. A significant association was detected between Ki67 staining and tumor invasion of the brain parenchyma. In comparison, the brains of mice bearing vector control U251 cells displayed a clear delineation of the margins of the tumor, with little invasion of contiguous brain parenchyma (Figure 2G). Our results indicate that overexpression of MFRN1 is associated with reduced OS and tumor aggression in GBM.

### 3.3. MFRN1 Overexpression Increases Mitochondrial Iron Level and Activity of ETC Complexes

**Figure 3:**
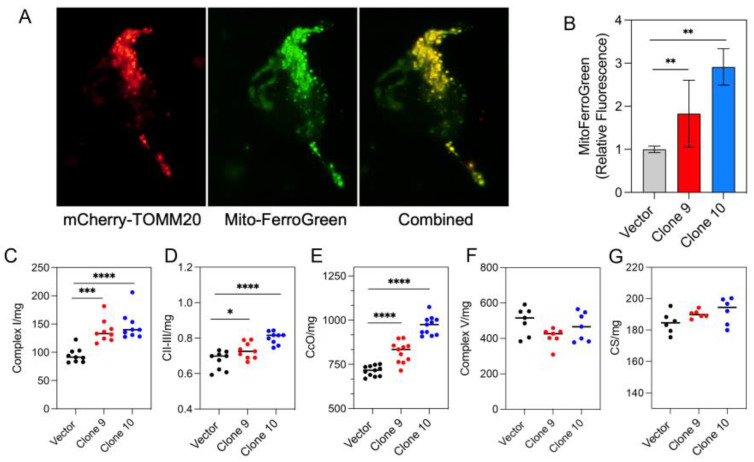
MFRN1 overexpression increases mitochondrial iron levels and the activity of ETC complexes. (**A**), MFRN1-overexpressing U251 cells were transfected with mCherry-TOMM20-N-10 (left panel) and stained with Mito-FerroGreen (middle panel) to confirm the mitochondrial localization of the detected iron (combined, right panel). (**B**), Quantification of the fluorescence intensity of the Mito-FerroGreen signal in MFRN1-overexpressing and vector-control expressing cells. (**C**–**G**), Activity of mitochondrial complex I (**C**), complexes II–III (**D**), CcO (**E**), citrate synthase (**F**), and complex V in MFRN1-overexpressing and vector control-expressing U251 cells. Graphs represent the mean ± SEM from duplicate determinations from at least three independent experiments. * *p* < 0.05, ** *p* < 0.01, *** *p* < 0.001 and **** *p* < 0.0001 calculated using one-way ANOVA followed by Tukey’s multiple comparison test or unpaired, two-tailed Student *t*-test.

To determine whether upregulated expression of MFRN1 affects mitochondrial iron content in glioma cells, we treated control and MFRN1-overexpressing cells with Mito-FerroGreen, a fluorescent mitochondrial-targeted iron probe that labels ferrous iron [33]. Transfection of MFRN1-overexpressing cells with mCherry-TOMM20-N-10, a plasmid that targets the mitochondrial outer membrane [38,39], revealed nearly complete colocalization with Mito-FerroGreen, confirming the specificity of Mito-FerroGreen for mitochondrial iron. Quantitative flow cytometry analysis showed a significant increase in fluorescence intensity in MFRN1-overexpressing cells (2-fold and 3-fold in clone 9 and clone 10, respectively) compared to vector control cells (Figure 3B), indicating a significant increase of mitochondrial iron in the MFRN1-overexpressing cells.

The transport of iron to the mitochondria is crucial for the synthesis of heme and Fe-S clusters and thus affects ETC function. Specifically, Fe-S clusters are required for the assembly, stability and function of mitochondrial ETC complexes I, II and III [40], whereas heme is required for the assembly and proper functioning of CcO (also known as complex IV) [41]. Therefore, we further investigated whether the increased incorporation of iron into the mitochondria through MFRN1 overexpression altered the activity of these ETC complexes. MFRN1 overexpression significantly increased the activity of complexes I–III and CcO (Figure 3C–E) but did not affect the activity of complex V (Figure 3F) and citrate synthase (Figure 3G), which do not require iron for assembly or function. These results suggest the importance of MFRN1 in the maintenance of mitochondrial iron homeostasis and mitochondrial function.

### 3.4. MFRN1 Overexpression Promotes Resistance to HNE-Induced Protein Damage

**Figure 4:**
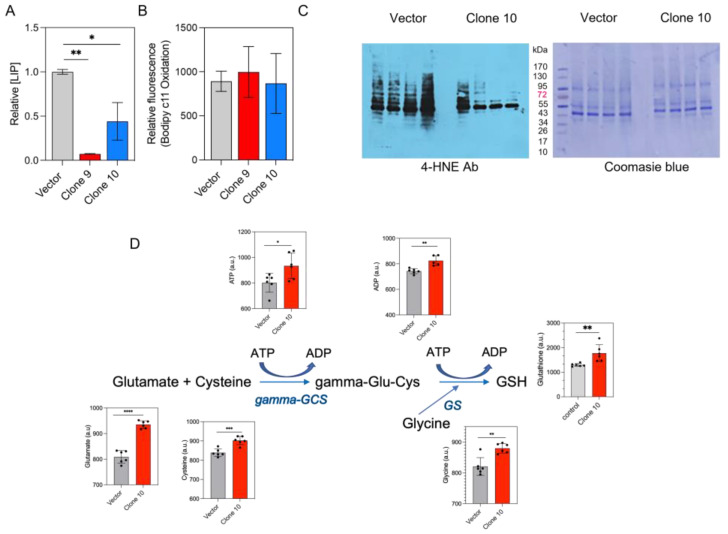
MFRN1 overexpression protects glioma cells from 4-HNE-induced damage. (**A**), Level of labile iron pool in vector control-transfected and MFRN1-overexpressing U251 cells; (**B**), Relative levels of lipid peroxidation in vector control-transfected and MFRN1-overexpressing cells, assessed with BODIPY C11; (**C**), Representative images depicting Michael adducts on proteins (left immunoblot) and total protein detection (right gel) before and after 1 h of 4-HNE treatment at the indicated doses in vector control-transfected and MFRN1-overexpressing cells. (**D**), Bars depict the levels of key metabolites involved in the glutathione pathway in vector control-transfected and MFRN1-overexpressing cells. Results of six independent measurements. * *p* < 0.05; ** *p* < 0.01; *** *p* < 0.001 and **** *p* < 0.0001 calculated using one-way ANOVA followed by Tukey’s multiple comparison test or unpaired, two-tailed Student *t*-test. a.u., arbitrary units. GCS, glutamyl cysteine synthetase; GS, glutathione synthase; GSH, reduced glutathione.

We recently demonstrated that the exposure of U251 cells to fractionated radiation causes an increase in CcO activity, which in turn increases the demand for mitochondrial iron, thus leading to mitochondrial iron loading and, consequently, reducing the labile iron pool and the level of lipid peroxidation [12]. As MFRN1 overexpression increases the level of mitochondrial iron and the activity of ETC complexes, we next investigated the impact of MFRN1 overexpression on the cellular labile iron pool. In agreement with our previous results, MFRN1 overexpression not only increased the level of mitochondrial iron (Figure 3A,B) but also depleted the LIP in U251 cells (Figure 4A) (*p* < 0.05). Cellular labile iron can promote lipid peroxidation [42,43,44] and downstream cellular damage; however, the level of lipid peroxidation was similar in these cells (Figure 4B).

Despite the apparent lack of effect on lipid peroxidation, however, MFRN1 overexpression completely prevented the dose-dependent increase in protein adduct formation induced by the 4-HNE treatment of the cells (Figure 4C). Conjugation with glutathione is a primary mechanism by which HNE-protein adducts are converted to less reactive species [45]. Therefore, we investigated whether MFRN1 overexpression affects glutathione metabolism. Metabolites involved in glutathione synthesis, including cysteine, methionine, glutamate and ATP, as well as glutathione, were upregulated in the MFRN1-overexpressing cells (Figure 4D), which could explain the lack of 4-HNE-mediated Michael adduct formation in these cells.

### 3.5. Inhibition of the Glutathione Pathway Induces 4-HNE-Mediated Protein Damage in MFRN1-Overexpressing Cells

**Figure 5:**
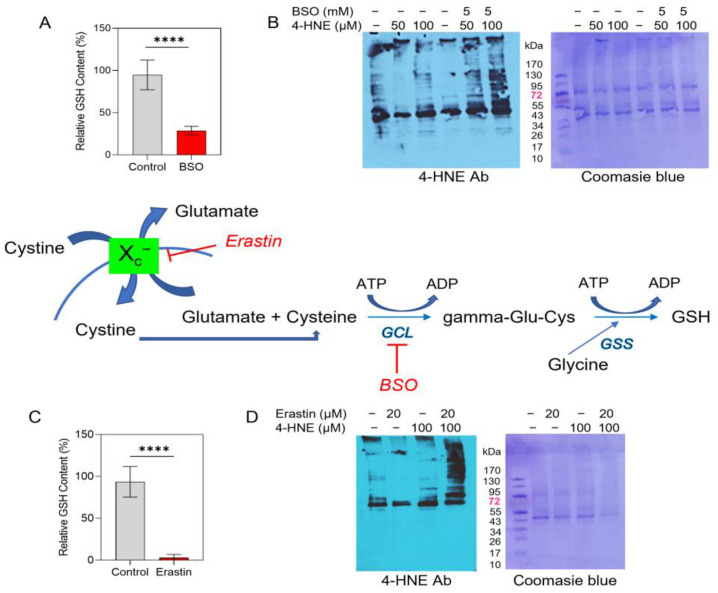
Inhibition of glutathione production facilitates 4-HNE-mediated protein damage in MFRN1-overexpressing glioma cells. (**A**), Quantification of intracellular GSH content in MFRN1-overexpressing U251 cells treated with 5 mM BSO for 24 h. (**B**), Representative images depicting the effects of BSO pretreatment (5 mM, 24 h) on the formation of 4-HNE-induced Michael adducts on proteins (left immunoblot) and total protein detection (right gel) in MFRN1-overexpressing cells. (**C**), Quantification of intracellular GSH content in MFRN1-overexpressing cells treated with 20 µM erastin for 24 h. **(D)** Representative images depicting the effects of erastin pretreatment (20 µM, 24 h) on the formation of 4-HNE-induced Michael adducts after 1 in MFRN1-overexpressing cells. **** *p* < 0.0001 calculated using one-way ANOVA followed by Tukey’s multiple comparison test or unpaired, two-tailed Student *t*-test.

To assess this possibility, we tested whether depleting the intracellular pool of glutathione could promote Michael adduct formation in the context of MFRN1 upregulation. We first investigated the effects of BSO, an agent that depletes endogenous glutathione [46] by inhibiting γ-glutamyl cysteine synthetase [47]. Exposure to 5 mM BSO for 24 h [30] led to a 3.5-fold decrease in intracellular GSH in MFRN1-overexpressing cells (Figure 5A). Significantly, BSO pretreatment enhanced the 4-HNE-induced adduction of many proteins of different sizes (Figure 5B). We further confirmed the involvement of the GSH pathway using erastin, an inhibitor of system xc transporter [48] that blocks the incorporation of the GSH precursor cysteine. Pretreatment with 20 µM erastin for 24 h [49,50] significantly reduced (<30 fold) the level of GSH in MFRN1-overexpressing cells (Figure 5C) and increased 4-HNE-induced protein adduction in MFRN1-overexpressing cells (Figure 5D). These results suggest that upregulation of MFRN1 in glioma cells protects against 4-HNE-dependent oxidative damage of proteins by upregulating cellular GSH levels.

## 4. Discussion

Mitochondrial ROS-induced cell damage underlies tumor progression and is crucial for the cytolytic effects of therapy in many cancers, including GBM [28,30,36,51,52,53,54], and mitochondrial iron is required to facilitate the assembly and function of the ETC complexes that are largely responsible for ROS production. However, the role of mitochondrial iron transporters in GBM progression and response to therapy is largely unknown. Recently, we discovered that radiation-induced resistance to therapy in glioma cells involves a decrease in cytosolic labile iron and an increase in mitochondrial ETC complex activity [12], suggesting the importance of mitochondrial iron transport. Our current findings reveal that the expression of the mitochondrial transporter MFRN1 is upregulated in GBM tumors and correlates with poor patient survival. Mechanistically, MFRN1-mediated mitochondrial import of labile iron facilitates greater mitochondrial ETC complex activity and promotes tumor progression and aggression in glioma cells. To our knowledge, this is the first study to show that the expression of MFRN1 regulates mitochondrial iron homeostasis in GBM and correlates with patient survival.

As mitochondrial iron importers, MFRN1 and MFRN2 regulate cytosolic and mitochondrial iron homeostasis [13,20,22]. The physiologic and pathophysiologic relevance of these proteins is still being defined, but the altered expression of each has been linked to iron homeostasis dysregulation and tumor progression or treatment resistance in some cancers [22]. For example, in vitro and in vivo evidence has suggested that MFRN1 regulates mitochondrial iron-induced ferroptosis in hepatocellular carcinoma cells and decreased expression of MFRN1 in these cells promotes cell survival and tumor growth [54]. Of particular relevance to our study of GBM, MFRN2 expression was found to be essential for ROS-mediated cytotoxicity in glioma cells treated with arsenic-trioxide [55]. Overall, however, it is becoming clear whether MFRN1 and MFRN2 are dysregulated and how this affects tumor growth and survival may differ by cell type [22,55,56,57]. For example, upregulation of MFRN2 in head and neck cancer cells confers sensitivity to radiation by a mechanism involving the accumulation of mitochondrial iron and the promotion of mitochondrial dysfunction [56]. Conversely, it has been reported that the expression of MFRN2 expression promotes the cytotoxicity induced by arsenic-trioxide in GBM [55]. Similarly, depletion of MFRN1 drives the acquisition of radioresistance in HeLa and oral squamous cells [58]; however in vitro and in vivo studies demonstrated that downregulation of MFRN1 promotes cell survival and tumor growth in hepatocellular carcinoma cells [54,59].

Our analysis of TCGA data revealed that MFRN1 is upregulated and MFRN2 is downregulated in GBM tumors, but only the expression of MFRN1 correlates with patient survival. Therefore, we focused our study on the cellular mechanisms affected by MFRN1 upregulation in glioma cells. Our data from U251 cells indicate that constitutive upregulation of MFRN1 increases the rate of cell proliferation and anchorage-independent cell growth, which is a hallmark of the aggressive tumor phenotype. This aggressive tumor phenotype was confirmed by the reduced survival of mice bearing MFRN1-overexpressing U251 cells. Furthermore, histopathologic analysis of the tumors from these mice indicated that the upregulation of MFRN1 enhanced the invasive or migratory nature of gliomas. Interestingly, these findings in GBM fit with a report showing that MFRN1 and MFRN2 are required for the increased proliferation of osteosarcoma cells exposed to excess iron but contrast with the report that reduced expression of MFRN1 in hepatocellular carcinoma cells promotes cell survival and tumor growth [60], further demonstrating the importance of thoroughly investigating the function of the mitoferrins by cancer cell type and conditions.

The pathologic effects of MFRN1 overexpression correlated with an increase in mitochondrial iron levels and the activity of ETC complexes in glioma cells. Although the role of mitochondrial iron homeostasis in cancer progression and survival has not been studied extensively, MFRN1 and mitochondrial iron homeostasis appear to be crucial in normal physiology and are dysregulated in several diseases [61,62,63,64,65]. In particular, the availability of iron is central to the mitochondrial biosynthesis of Fe-S cluster proteins [66]. Our data also support the essential role of mitochondrial iron in cellular proliferation. In agreement with this result, Seguin et al. reported that MFRN1 and MFRN2 are required for mitochondrial iron import to maintain mitochondrial and cellular iron homeostasis during the active proliferation of normal mammalian cells [60]. Furthermore, Ni et al. showed that iron supplementation increases the proliferation rate of osteosarcoma cells by increasing the mitochondrial iron level in a manner dependent on MFRN1 and MFRN2 expression [57], while Sandoval-Acuna et al. showed that depleting mitochondrial iron abrogates breast cancer invasion in vitro and metastasis in vivo [15].

The increase in mitochondrial iron concentration in MFRN1-overexpressing glioma cells correlated with a decrease in the labile iron pool, suggesting the transport of labile iron into the mitochondria. Altered expression of MFRN1 has also been shown to affect the labile iron pool in skeletal muscle cells of older adults as well [67]. Because it is not bound to transferrin or other ligands, labile iron can readily participate in the Fenton-type reactions that generate highly toxic free radicals (RO·or ROO) [68,69] that in turn enhance lipid peroxidation [70,71,72,73] and, consequently, the production of reactive aldehydes such as 4-HNE that form Michael-type adducts [45,74]. Interestingly, the decrease in labile iron observed in MFRN1-overexpressing cells was not accompanied by a decrease in lipid peroxidation, yet MFRN1-overexpressing cells were still protected from 4-HNE protein adduction. Perhaps explaining this protection in the absence of a change in lipid peroxidation, GSH was upregulated in MFRN1-overexpressing cells. Confirming the protective effect of the increase in GSH, blocking the glutathione pathway allowed the formation of 4-HNE protein adducts. Previous studies have revealed the importance of glutathione conjugation in the detoxification of 4-HNE to less reactive chemical species [11]. Our results now suggest a link between MFRN1-regulated mitochondrial iron metabolism and the glutathione pathway, as well as the relationship thereof to GBM progression and aggressiveness.

Although more investigation is required to further understand the underlying mechanism, this link also indicates that targeting mitochondrial iron metabolism could be an effective strategy for enhancing therapeutic efficacy in GBM. The modulation of iron metabolism using iron chelators has been shown to inhibit cancer cell growth and progression in several studies [74,75,76]. Indicating the relevance of mitochondrial iron metabolism, specifically, Sandoval-Acuna et al. showed that depletion of mitochondrial iron suppresses tumor growth and metastasis in breast cancer [15]. We have also shown that treatment with VLX, which specifically chelates mitochondrial iron, restores radiosensitivity in GBM cells that become radioresistant in response to fractionated radiation [12]. To further assess the therapeutic potential of targeting MFRN1, our future studies will determine whether the increased expression of MFRN1 also contributes to the radiation-induced effects on labile iron and ETC function that we found to promote radioresistance in glioma cells. While we did not find a correlation between the tumor expression of MFRN2 and patient survival, it will also be important to assess the role of MFRN2 in the development of GBM radioresistance, considering that MFRN2 has been implicated in the ROS-mediated cytotoxicity induced in glioma cells by arsenic trioxide [55].

## 5. Conclusions

In conclusion, the findings in this study provide evidence that MFRN1 expression is closely associated with GBM proliferation in vivo and in vitro. Moreover, these findings suggest that further investigation of MFRN1 and mitochondrial iron metabolism could lead to new strategies for targeting therapy-resistant GBM.

## Data Availability

The data are contained within the article or Supplementary Materials.

## Supplementary Materials

The following supporting information can be downloaded at: https://www.mdpi.com/article/10.3390/antiox12020349/s1, Original images of western blots (Figure 2B, Figure 4C, Figure 5B,D)
