# Supplementary figures and images for: Mitoferrin-1 Promotes Proliferation and Abrogates Protein Oxidation via the Glutathione Pathway in Glioblastoma
Source: Antioxidants (Basel). 2023 Feb 1;12(2):349. doi: 10.3390/antiox12020349 (PMC9952016; doi:10.3390/antiox12020349)

Figure 2C

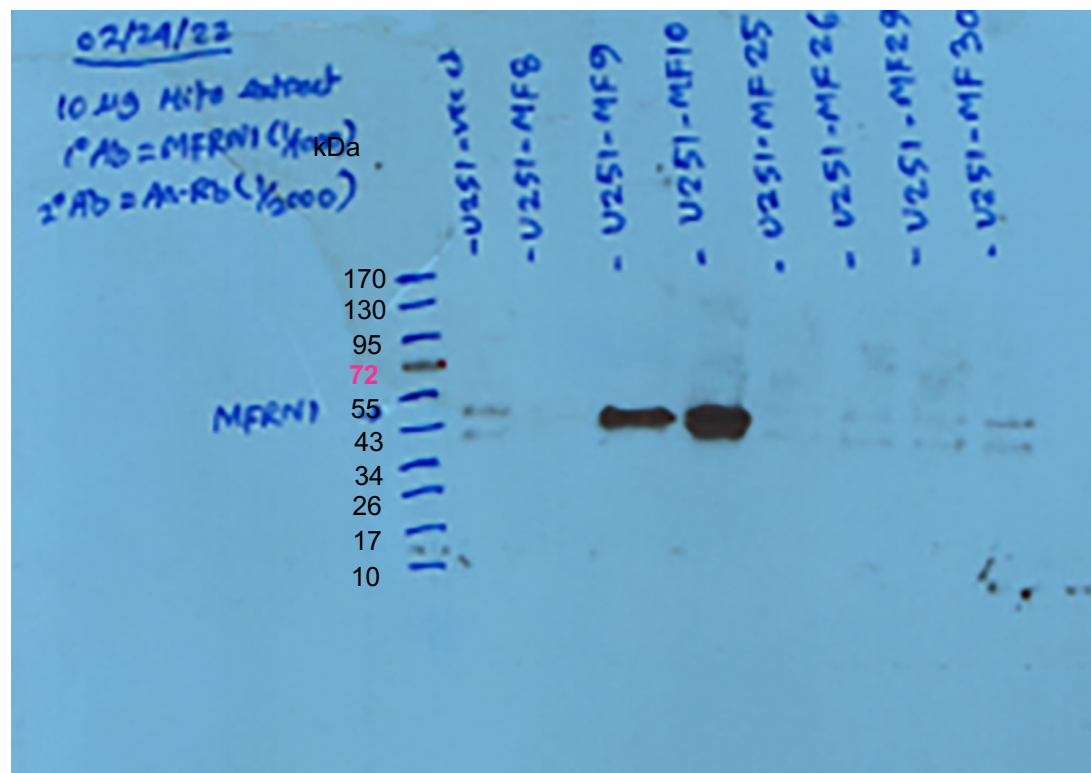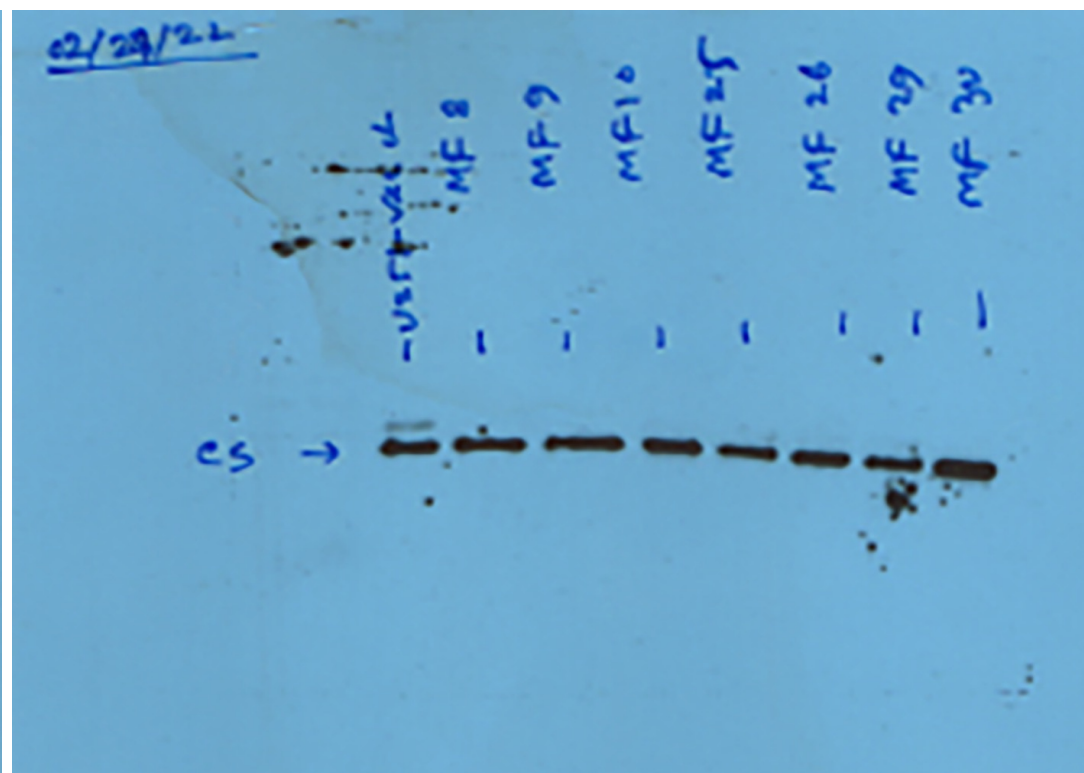

Figure 4C

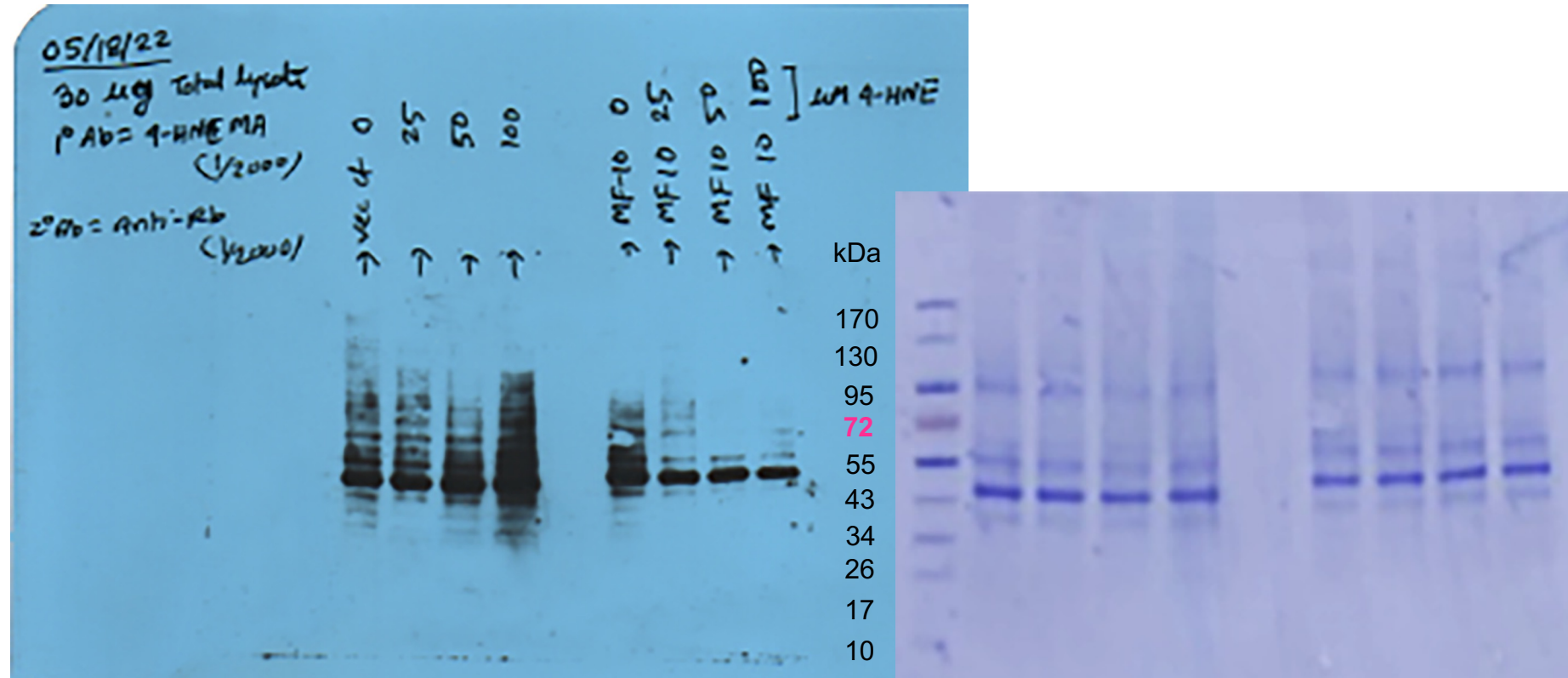

Figure 5B

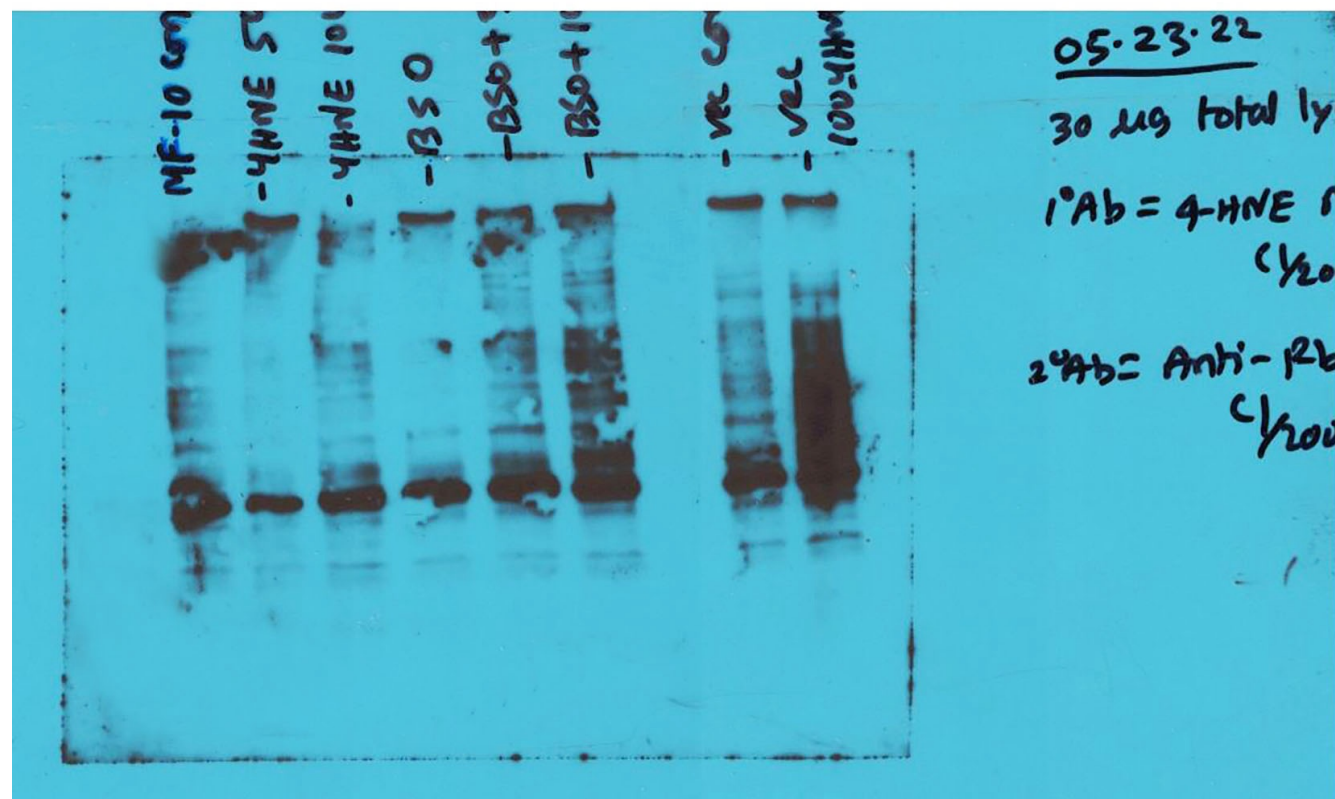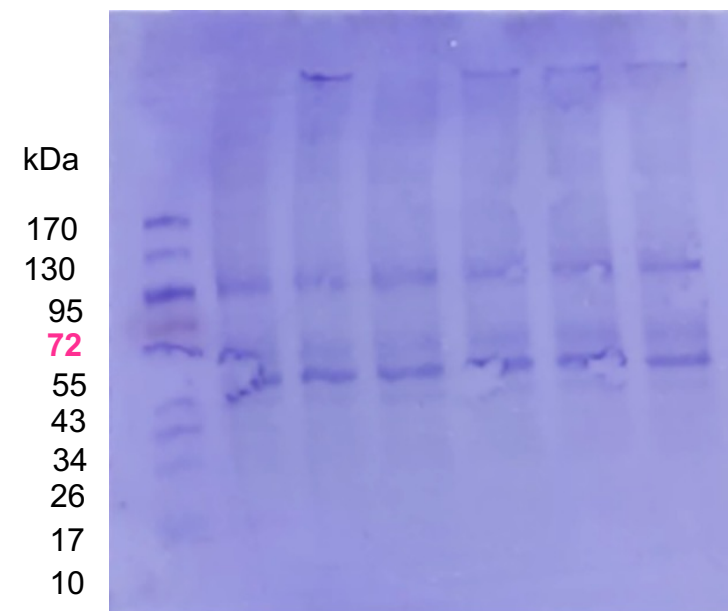

Figure 5D

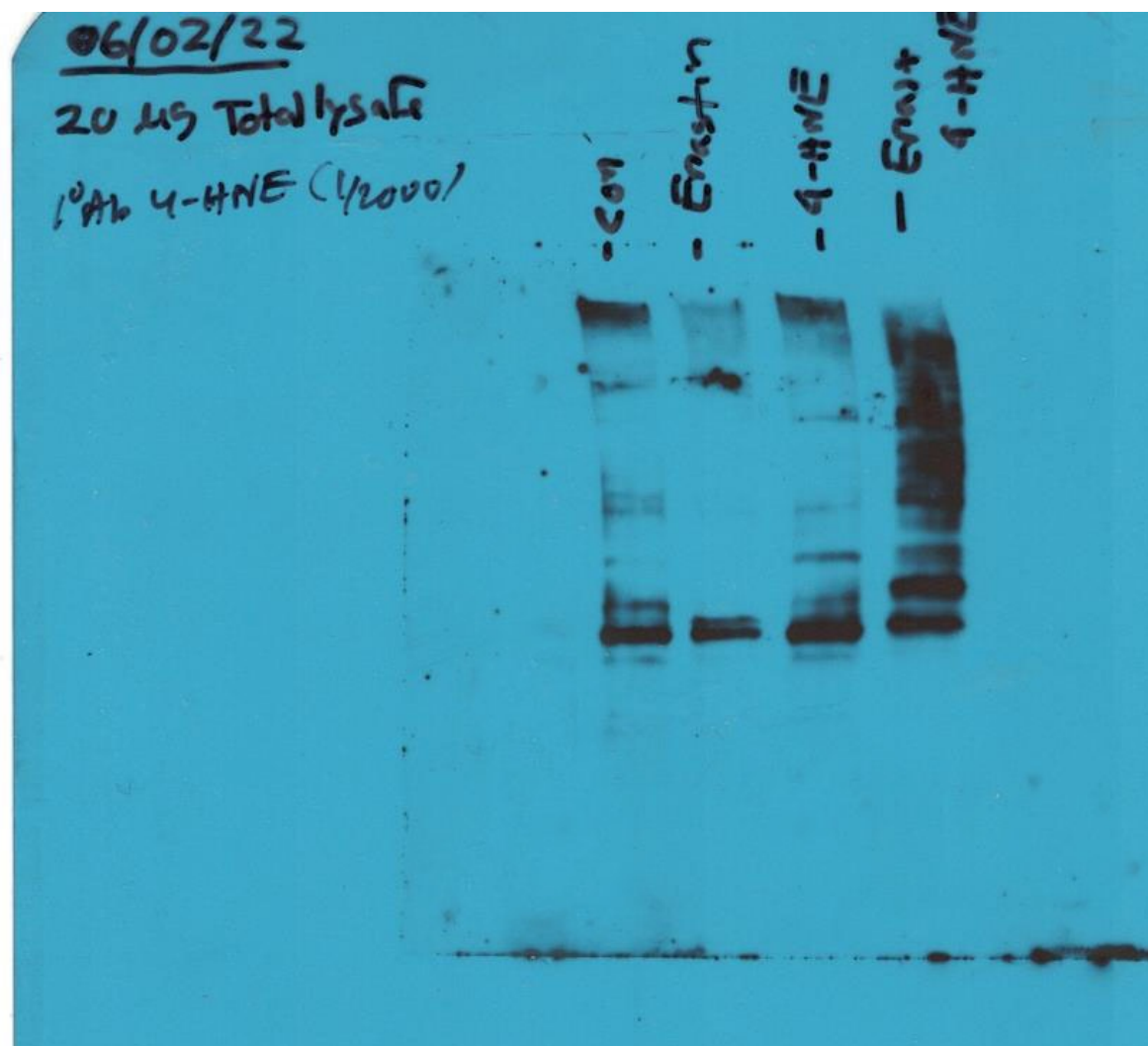

kDa

170

130

95

72

55

43

34

26

17

10

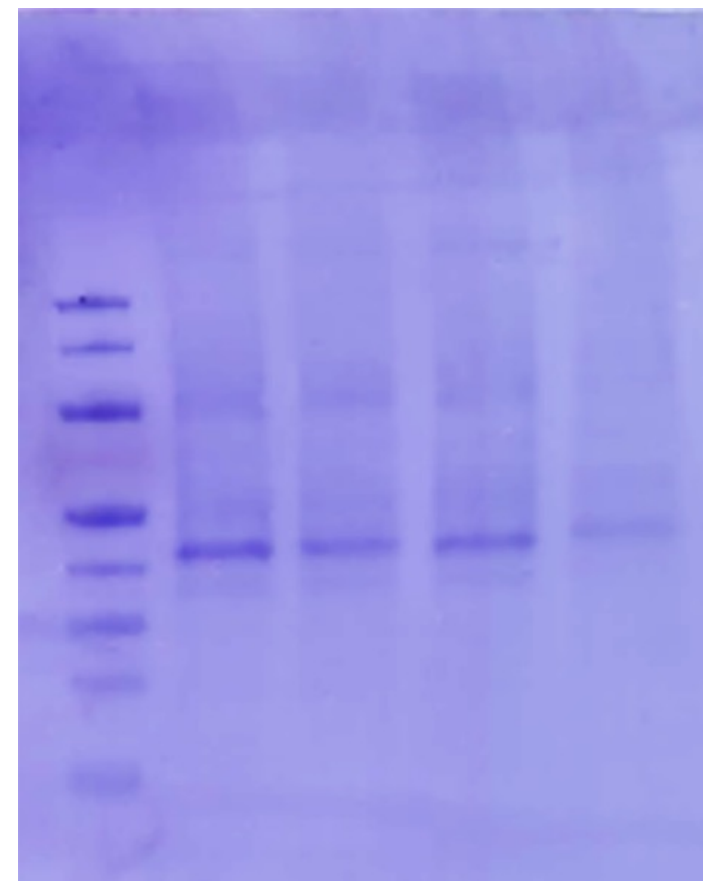

Supplement: Supplementary file 1 [file antioxidants-12-00349-s001.zip › antioxidants-2195639-supplementary.pdf]
